# Herd and animal level seroprevalence and associated risk factors of small ruminant brucellosis in the Korahey zone, Somali regional state, eastern Ethiopia

**DOI:** 10.3389/fvets.2023.1236494

**Published:** 2023-09-19

**Authors:** Ahmed Mohammed Hussen, Fikadu Alemu, Ahmed Hasan Hussen, Abdimalik Hussein Mohamed, Haben Fesseha Gebremeskel

**Affiliations:** ^1^College of Veterinary Medicine, Jigjiga University, Jigjiga, Ethiopia; ^2^School of Veterinary Medicine, Wolaita Sodo University, Wolaita Sodo, Ethiopia

**Keywords:** brucellosis, goat, risk factors, seroprevalence, sheep

## Abstract

**Introduction:**

Brucellosis is a zoonosis of major public health and economic importance that is endemic in livestock in Ethiopia with varying levels of seroprevalence.

**Methods:**

A cross-sectional study was carried out to determine the individual and herd-level seroprevalence of brucellosis in small ruminants in the Korahey zone of the Ethiopian Somali Region. A total of 324 sera from 63 herds of small ruminants were collected randomly using a multistage sampling technique and the sera were tested using the Rose Bengal Plate Test, and seropositive reactors were confirmed by the Complement Fixation Test.

**Results and discussion:**

The seroprevalence of brucellosis at the herds and the individual level was 6.35% (95% CI: 0.0–13%) and 1.23% (95% CI: 0.0–2%), respectively; with 1.4% in goats and 0.9% in sheep. Moreover, predicted variables like age group, parity, history of abortion, fetal membranes, herd size, ownership of other livestock species, contact with wild animals in the past year, the introduction of new animals in the past year, and lending of breeding males in the past year were not significantly associated (*p* > 0.05) with *Brucella* seropositivity at individual and herd level seroprevalence during multivariable logistic regression analysis. Pastoral community awareness regarding the public health impact of brucellosis and the promotion of an intersectoral One Health approach for the effective control of brucellosis is recommended.

## 1. Background

Brucellosis is a bacterial zoonotic disease caused by the genus *Brucella* that causes reproductive problems such as abortion, retained fetal membranes, and the birth of weak offspring, as well as orchitis and epididymitis in male animals, which is frequently followed by sterility (1). There are 12 known *Brucella* species that cause brucellosis at this time (2) and six of them, are known to be pathogenic to humans: *B. abortus*, *B. canis*, *B. inopinata*, *B. melitensis*, *B. pinnipedialis*, and *B. suis* (2, 3). This taxonomic classification is primarily based on differences in host preference and pathogenicity that can be attributed to different proteomes, as demonstrated by specific outer-membrane protein markers (4, 5). Around the world, *Brucella* species that infect both humans and animals are frequently discovered in the interface of the human-animal ecosystem, where strong interactions exist between people, livestock, and wildlife in the same area (6, 7).

*Brucella abortus* causes abortion, stillbirth, and weak calves in cattle, with abortions typically occurring during the third trimester of pregnancy. *B. melitensis* is the most common species in developing countries in small ruminants and is linked to clinically visible diseases in humans (8). *B. melitensis* can cause abortion, retained placenta, orchitis, and epididymitis in goats. Abortions in goats are most common during the fourth month of pregnancy (9). Men’s clinical manifestations of the disease include weakness, fever, excessive sweating, especially at night, weight loss, and generalized body aches (10). Swelling in the testes and burning micturition caused by orchitis and urethritis, respectively, are unusual symptoms of the disease in men (5, 11). The disease has also been reported in wild and marine mammals, as well as birds, in recent years. Another epidemiological concern is the presence of brucellosis in wild animals, with the potential for continuous transmission to domestic animals and from them to humans (12).

Prevalence studies for brucellosis have been carried out in various parts of the country. Brucellosis in animals and humans has been reported in various parts of Ethiopia, most notably in cattle in both intensive and extensive management systems (13–17). The disease has also been reported among small ruminants in pastoral areas of the country. In a study conducted in the Tallalak district of the Afar region, a prevalence of 13.7% in sheep and goats was reported, with the prevalence being higher in goats (15.4%) than in sheep (10.6%), as reported by Wedajo et al. (18), whereas in the Somali region, Mohammed et al. (19) reported a seroprevalence of 1.37% among small ruminants in three woredas of the Jigjiga zone.

Unpasteurized milk products, infected placental material, aborted fetuses, or infected animals, which can shed a variety of bacteria after abortion, are other ways in which this infection in humans can occur in endemic countries (20). Pastoralist communities are more likely to contract brucellosis and other zoonotic diseases than other communities that do not have the same level of association with animals due to their close physical proximity to livestock and their reliance on animal products (19, 21).

The prevalence of brucellosis in pastoralist areas is thought to be influenced by several variables, including grazing patterns, management techniques, and the age and sex composition of herds (22, 23). Additionally, for a variety of reasons, pastoralists do not isolate or get rid of animals that may be infected with Brucellosis, which raises the risk of transmission to healthy animals. Female sheep and goats are kept in the herds for a longer period than males and are sold as soon as they mature. Unlike males, infected females shed the bacteria more frequently and may contribute to the likelihood of brucellosis in pastoral areas. Other factors contribute to the prevalence of brucellosis in pastoralist areas, such as the consumption of unpasteurized milk; unsafe handling and improper disposal of potentially infective materials, such as aborted fetuses, fetal membranes, and bodily fluids, which may contain concentrations of the bacteria; and a lack of awareness about zoonotic risks and methods of transmission (4, 16, 17, 20).

Nonetheless, the extent of the disease and its impact on pastoralist health are understudied, at least in the context of the proposed study area: the Korahey zone. Similarly, no research has been done on the risk factors associated with disease occurrence or the existing knowledge, attitudes, and practices in pastoralist areas that may play a role in zoonotic transmission. Therefore, the objective of this study was to estimate seroprevalence and associated risk factors of brucellosis in small ruminants in selected districts of the Korahey zone, Somali regional state, Ethiopia.

## 2. Methods

### 2.1. Study area

**Figure 1:**
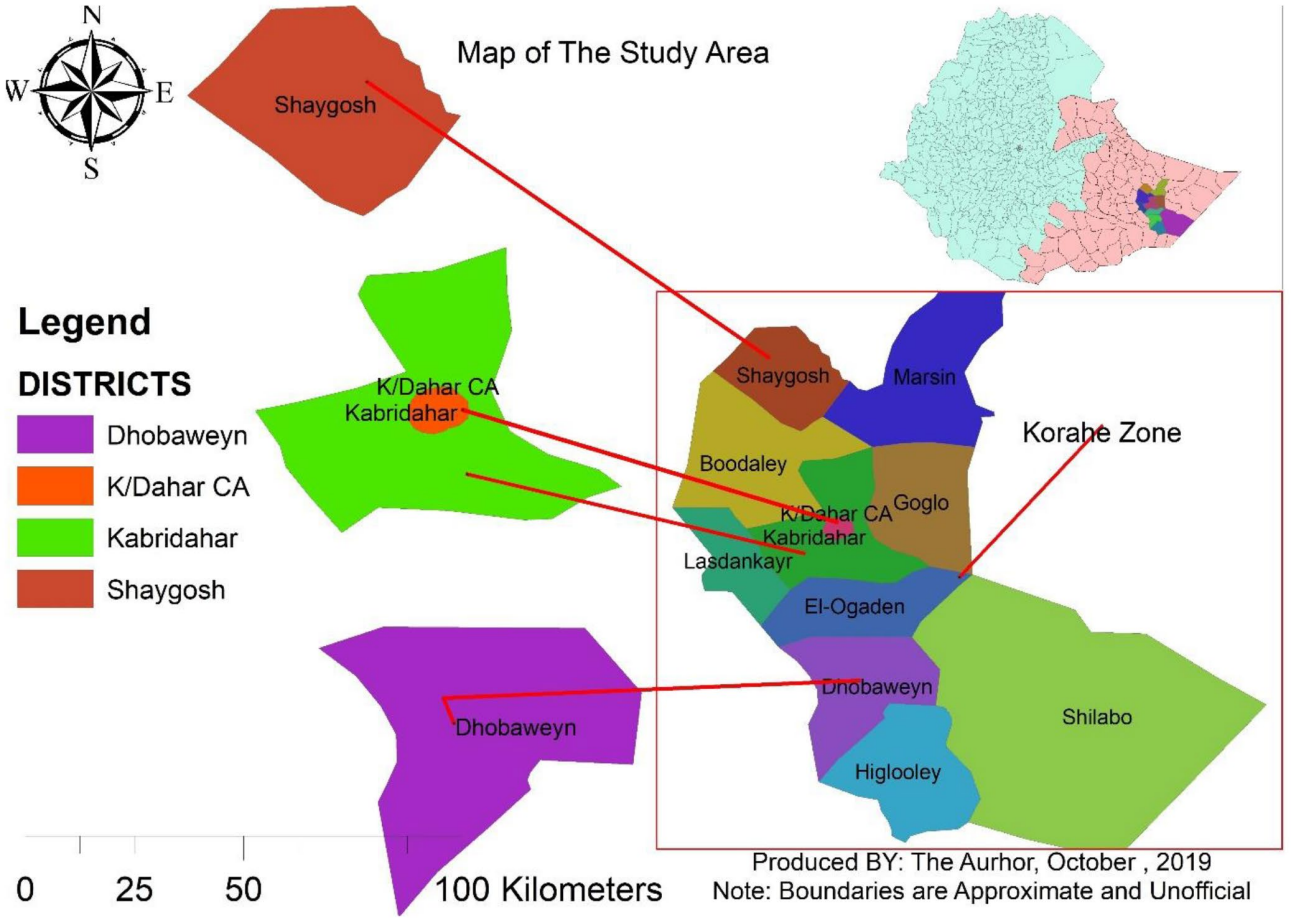
Map of the three study districts of the Korahey zone, Somali regional state, and eastern Ethiopia.

The study was conducted in three purposively selected districts of the Korahey zone, namely, Doboweyn, Kebridahar, and Sheygosh, located 470, 380, and 280 km, respectively, south of Jigjiga, the capital of the Somali Region of Ethiopia, which is situated approximately 630 km to the east of Addis Ababa. The Korahey zone is located in the south of the region and consists of 10 districts and one city administration; the zonal capital is Kebridahar. The zone has semiarid agroecology with a bimodal rainfall pattern. The main rainy season is *Gu* (April–June), and the second rainy season, *Deyr,* is received between September and November. The major production system in the zone is pastoralism, with some mixed livestock crop production practiced around Kebridahar. The livestock population of the zone is 3,576,492, consisting of 311,243 cattle, 1,323,491 goats, 1,265,585 sheep and 582,860 camels (24). Kebridahar is located at 6°44′N latitude and 44°16′E longitude and has a total of 799,367 of livestock population. Doboweyn is located at 6°41′N latitude and 43° 69′E longitude and has a total of 626,674 of livestock population. Sheygosh is located at 7° 41’ N latitude and 43° 56′ E longitude and has a total of 520,900 of livestock population (Figure 1).

### 2.2. Study population

There are approximately 1,323,491 goats and 1,265,585 sheep in the Korahey zone (25). The study population consisted of small ruminants kept under an extensive management system in three purposively selected districts of the Korahey zone. The study animals were indigenous Somali goats and black head Ogaden sheep. In addition, sheep and goats, which were above 6 months of age and had no history of vaccination against brucellosis, were included in the study.

### 2.3. Study design

A cross-sectional study design was used to estimate the seroprevalence and associated individual animal-level and herd -level risk factors for small ruminant brucellosis and to evaluate the knowledge, attitudes and practices of pastoralists toward brucellosis in selected districts of the Korahey zone, Somali region of Ethiopia.

### 2.4. Sampling method

A multistage sampling technique was employed. The pastoralist association was the primary sample, pastoralist families/herds the secondary sample and the individual animals the tertiary sample. The primary sample was selected purposively based on livestock population and accessibility, whereas the secondary and tertiary samples, herds and individual animals, were selected using a systematic random sampling technique.

Individual animals were sampled from the herds above using a systematic random sampling method by first putting the animals in a crush. Then, the animals were allowed to leave the fence one animal at a time. The herd’s owner was asked to randomly pick an animal from among the first 5, leaving the fence. Then, every k^th^ animal was selected; the value of k was determined based on the size of the herds being sampled and the number of animals sampled from each herd. Sheep and goats were sampled separately.

### 2.5. Sample size determination

The sample size was determined using Thrusfield (26) formula as follows:


$$n=\frac{1.96^{2}\mathrm{Pexp}\;\left(1-\mathrm{Pexp}\right)}{d^{2}}$$

where n = required sample size, Pexp = expected prevalence and d = desired absolute precision.

Accordingly, the estimated sample size was 23 for goats and 15 for sheep based on the expected brucellosis prevalence of 1.5% in goats and 1% in sheep in other parts of the Somali region by Mohammed et al. (19) and 0.05 desired absolute precision at the 95% level of confidence. However, to increase precision, the sample size was increased to 213 goats and 111 sheep. In total, 324 small ruminants (sheep and goats) were sampled using systematic random sampling (Table 1).

**Table 1:** Total number of small ruminants sampled from each kebele (peasant associations).

Woreda	Kebele	Total herds listed	Herds sampled	Animals sampled		
				Goat	Sheep	Total (% sampled)
Doboweyn	Doboweyn town 01 kebele	84	9	17	17	34 (10.5)
	Harano	112	9	20	14	34 (10.5)
	Jidhale	98	9	20	14	34 (10.5)
Kebridahar	01 Kebele, Kebridahar	71	9	23	13	36 (11.1)
	Bundada	131	9	33	9	42 (13)
	Dalad	117	9	26	8	34 (10.5)
Sheygosh	01 Kebele Sheygosh	66	9	19	19	38 (11.7)
	Harir	109	9	29	9	38 (11.7)
	Wijiwaji	96	9	26	8	34 (10.5)
	Total	884	63	213	111	324

Animals were sampled from nine purposively selected kebeles that were located in three study districts, i.e., three kebeles (peasant associations) per woreda (districts) based on livestock population and accessibility. The distribution of the herds across the kebeles and woredas was determined based on the estimated population size of the respective kebeles.

### 2.6. Blood sample collection, transportation, and storage

Approximately 10 mL of blood sample was collected from the jugular vein of each study animal using plain vacutainer tubes, needle holders, and needles. The blood sample from each animal was labeled and centrifuged at 10,000 rpm for 3 min, and sera were removed by siphoning them into sterile cryovials. The serum samples were then transported to the Jigjiga regional veterinary diagnostic and research laboratory in an ice box, where they were stored and kept at −20°C until serology procedures were performed.

### 2.7. Serological tests

**Figure 2:**
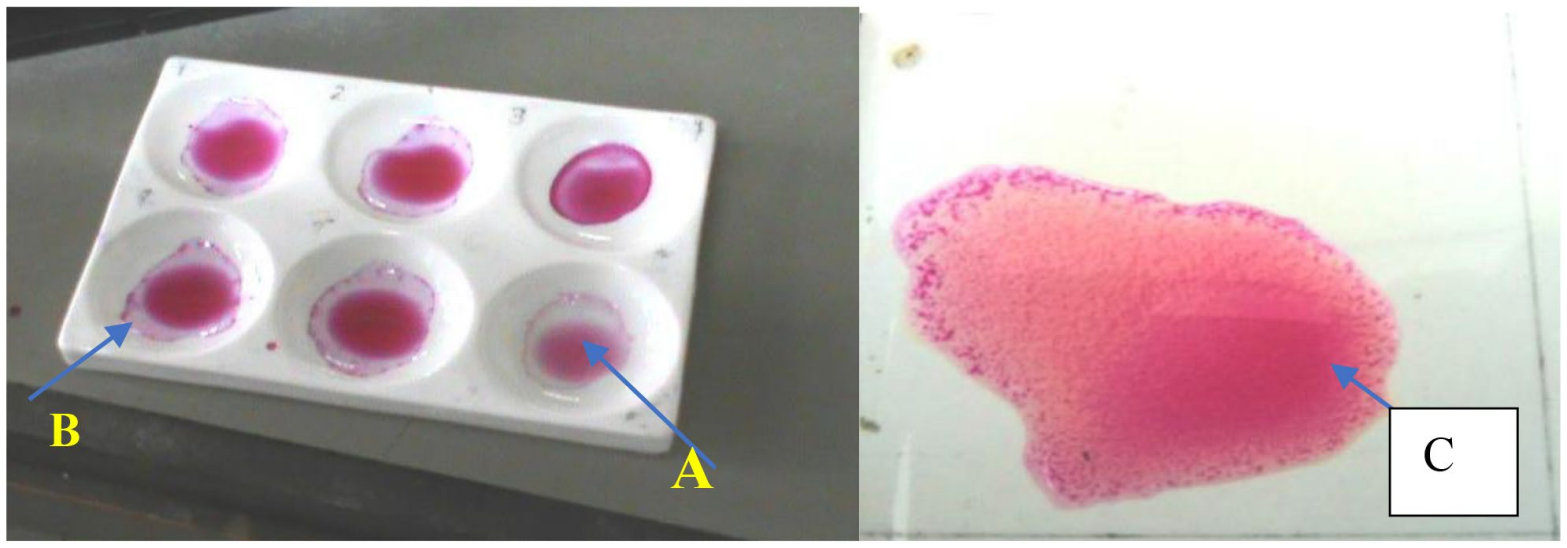
Plate Rose Bengal Plate Test showing positive and negative samples. A, positive sample; B, negative sample; C, positive control.

The screening procedure with RBPT was performed at Jigjiga Regional Veterinary Diagnostic and Investigation Laboratory and National Veterinary Institute (NVI) at Bishoftu, whereas CFT was conducted at NVI using test protocols as outlined by OIE (27) and the manufacturer’s specifications for the tests. All the samples that tested positive on the RBPT were tested with the complement fixation test (CFT) (Figure 2).

The CFT and RBPT test antigens (*Brucella abortus* strain 99), control sera, and other reagents were obtained from Atlas Medical, William James House, Cowley Rd. Cambridge Cb4, 4 WX and sensitized sheep red blood cells (SRBCs) were obtained from the NVI. The serum specimens were tested serially first using RBPT and then CFT for those that tested positive for RBPT. An animal was considered positive if the serum specimen tested positive on both RBPT and CFT, whereas a herd was considered positive if at least a single serum specimen from an animal within the herds tested positive on both RBPT and CFT. For RBPT, the Rose Bengal test antigen was prepared from the killed standard strain of *B. abortus* strain 99 and stained with Rose Bengal dye in an acidic buffer pH 3.65.

### 2.8. Questionnaire survey

A pretested structured questionnaire was used to collect information about potential factors associated with brucellosis seropositivity at animal and herd levels. The potential factors at the individual animal level included age, parity stage, and previous history of reproductive problems such as abortion, retained fetal membranes, orchitis, and epididymitis. At the herd’s level, the factors assessed were herd size, keeping other animals with sheep and goats, the introduction of new animals in the past year, abortion in the herds in the past year, contact with other small ruminant herds and wild animals, presence of calving/lambing/kidding pens, vaccination history and whether owners sought veterinary service or advice in the past year.

### 2.9. Data management and analysis

Serological data were entered into a Microsoft Excel Spreadsheet version 2016 (Microsoft Corporation) along with the corresponding data generated with the questionnaire. The statistical software package SPSS version 20 (SPSS Inc., IBM, Chicago, IL, United States) was used for data analysis. Descriptive statistics like frequency and proportion were employed for the description of the seroprevalence of the disease. A herd level and individual animal seroprevalence were calculated by dividing the number of positive test results by the total number of herds and animals sampled, respectively. Univariable analysis using Fisher’s exact test was used for the analysis of the association between individual animal-level and herds *Brucella* seropositivity and the potential factors. Furthermore, a multivariable logistic regression model was used to analyze risk factors of the disease that was found statistically significant when using univariable analysis and the results were reported by odds ratio using 95% confidence interval to assess the strength of the association. The final multivariable logistic regression model selection was based on *p* value (p value ≤0.25) and stepwise backward elimination procedure, dropping the least significant independent variable until all the remaining predictor variables were significant. The statistical significance level was set at 95% confidence level and 5% level of precision so that *p* ≤ 0.05 was considered significant.

## 3. Results

### 3.1. Herds characteristics

A total of 324 animals composed of 213 (65.7%) goats and 111 (34.3%) sheep were sampled. These animals were sampled from 63 herds from 9 kebeles (peasant associations) in Doboweyn, Kebridahar, and Sheygosh districts of the Korahey zone, Somali Region of Ethiopia. The 63 herds had a total of 4,026 animals, of which 2,512 were goats and 1,514 were sheep. The median sheep and goat herds’ size was 56 (range: 20–180 animals). One-third of the herds in the study (33.3%) had more than 60 heads of sheep and goats, and the remaining 66.7% had a herd size of 60 heads or below.

### 3.2. Seroprevalence of brucellosis at the individual animal and herd levels

#### 3.2.1. *Brucella* seroprevalence at the individual animal level by animal species

Of the 324 serum samples examined, 1.85% (95% CI: 0.0–3.0%) were positive for *Brucella* antibodies on RBPT. Of the samples positive for RBPT, 2.3% (95% CI: 0.0–4%) were from goats, and 0.9% (95% CI: 0–3%) were from sheep. Of the samples testing positive on RBPT, 1.23% (95% CI: 0.0–2%) tested positive on CFT, of which 1.4% (95% CI: 0.0–3%) were goats and 0.9% (95% CI: 0–3%) were sheep. In this study, the animal-based seroprevalence of small ruminants brucellosis in three district of the Korahey zone of Somali regional state was 1.85% by RBPT and 1.23% by combined RBPT and CFT. Thus, the overall seroprevalence was 1.23% and were taken for subsequent data analyses (Table 2).

**Table 2:** Seroprevalence of *Brucella* antibodies by test type and species.

Test	Species	No of examined animals	No. of positive sampled	Percentage (%)	95% CI
RBPT	Sheep	111	1	0.9	0.0–3.0
	Goat	213	5	2.3	0.0–4.0
	Total	324	6	1.85	0.0–3.0
CFT	Sheep	111	1	0.9	0.0–3.0
	Goat	213	3	1.4	0.0–3.0
	Total	324	4	1.23	0.0–2.0

#### 3.2.2. *Brucella* seroprevalence at the individual animal level by sublocation sampled

Based on CFT, the highest seroprevalence of 5.9% (95% CI: 2–14%) was recorded in the Dalad kebele of Kebridahar district, followed by 2.9% (95% CI: 3–9%) in the Harano kebele of Doboweyn district and then by 2.6% (95% CI: 3–8%) in the 01 kebele in Sheygosh town of Sheygosh district. In the remaining 6 locations, namely, 01 kebele of Kebridahar town and Bundada kebele of Kebridahar district; 01 kebele of Doboweyn town and Jidhale kebele of Doboweyn district and Harir and Wijiwaji kebeles of Sheygosh district, zero seroprevalences were recorded.

#### 3.2.3. *Brucella* seroprevalence at the herd level

Of the 63 herds sampled, the overall true herd seroprevalence was 6.35% (95% CI: 0.0–13%). None of the herds had more than one animal testing positive for *Brucella* species. All the herds sampled had a mixture of sheep and goats; hence, comparing herds’ seropositivity levels by species was deemed irrelevant.

### 3.3. Factors associated with brucellosis seropositivity at individual and herd levels

#### 3.3.1. Bivariate analysis for individual-level factors associated with brucellosis in sheep and goats

In determining the risk factors associated with individual animal *Brucella* seropositivity, several factors were examined in the bivariate analysis. These include species, sex, age, number of parties, previous history of abortion and/or retained fetal membrane, and previous history of orchitis and epididymitis.

In terms of sex, 276 (85.2%) were females, and 48 (14.8%) were males. All the seropositive animals were females, but there was no statistically significant difference in the brucellosis seroprevalence between the two sexes (*p* > 0.05).

The majority, 196/324 (60.5%), of the sampled animals were between 3 and 4 years old, and only 18 (5.5%) were older than 4 years. A significant difference (*p* = 0.001) was observed between the different age categories with regard to *Brucella* seropositivity, with the highest prevalence being in the >4-year-old age category (16.7%), followed by the category aged 3–4 years (0.5%). None of the animals in the age range of 1–2 years tested positive for *Brucella*.

Of the female animals in the sample, 258/276 (93.4%) gave birth at least once, with 13 (4.7%) of them being primiparous, giving birth only once. Most of the female animals (84.8%) gave birth between 2 and 4 times. Only 11 females (4%) gave birth more than four times. The highest seroprevalence was observed in the category of female animals that gave birth more than 4 times, with 3 of the 11 female animals in this category (27.3%) showing seropositivity with CFT. This was followed by the females that gave birth between 3 and 4 times, in which a seropositivity prevalence of 0.6% was recorded.

A quarter, 69/276 (25%), of the female animals sampled had a history of abortion at least once in the past, whereas 49/276 (17.8%) of female animals experienced retained fetal membranes. Regarding male animals, 11/48 (23%) had a history of experiencing either orchitis or epididymitis, but this study did not find any seropositive males. All the seropositive animals had a previous history of abortion and retained fetal membranes, and it was found that there was a significant difference (*p* < 0.05) between animals with a history of previous abortion and those without a previous history of abortion (Table 3).

**Table 3:** Univariate analysis of seropositivity of *Brucella* with associated risk factors at individual animal level (*n* = 324).

Variable	Category	*Brucella* seropositivity n (%)	Negative n (%)	χ^2^	*p*
Species	Goats	3 (1.4)	210 (98.6)		0.69
	Sheep	1 (0.9)	110 (99.1)	0.154	
Sex	Female	4 (1.4)	272 (98.6)		0.401
	Male	0	48 (100)	0.704	
Age category	≤2 years	0	110 (100)		<0.0001*
	3–4 years	1 (0.5)	195 (99.5)	37.375	
	>4 years	3 (16.7)	15 (83.3)		
Number of parity	0–2 times	0	102 (100)		<0.0001*
	3–4 times	1 (0.6)	162 (99.4)	53.655	
	5–6 times	3 (27.3)	8 (72.7)		
Previous history of abortion	Yes	4 (5.8)	65 (94.2)	14.967	0.001*
	No	0	207 (100)		
Retained fetal membrane	Yes	4 (8.2)	45 (91.8)	22.730	<0.0001*
	No	0	227 (100)		
Orchitis and epididymitis	Yes	0	11 (100)	0.704	0.703
	No	0	37 (100)		

*Indicates variables with a statistically significant values. OR, odds ratio; CI, confidence interval.

#### 3.3.2. Bivariate analysis of seropositivity of *Brucella* with herd-level risk factors in sheep and goats

In determining the risk factors associated with herds *Brucella* seropositivity, several factors were examined in the bivariate analysis. These included herds’ size, ownership of other livestock species, ownership of calving/kidding/lambing pens, contact with other herds and contact with wildlife, history of abortion in the herds, the introduction of new animals, lending/borrowing of male animals, and veterinary service-seeking behavior of the owner. All but the last factor, i.e., veterinary service-seeking behavior, was considered a risk factor, while the latter variable was treated as a protective factor.

One-third of the herds (21/63, 33.3%) had more than 60 heads of sheep and goats, and the remaining herds had a maximum herd size of 60 sheep and goats. The herds with a size of more than 60 animals had almost 7 times higher odds of having positive reactors for *Brucella* compared to those herds with smaller herds sizes, but this was not statistically significant (OR = 6.833; 95% CI: 0.665–70.235, *p* = 0.106).

Of the 63 sampled sheep and goat herds, 25 (39.7%) are kept together with other livestock species, as the owner also keeps other species of livestock apart from goats and sheep, and the other 38 (60.3%) households possess only small ruminants. Almost all of the respondents, 24/25 (96%), who also own other livestock species raise the different herds separately, and only one respondent said that they house the different species of animals together. Most of the other livestock species kept in the area are camels, cattle, and donkeys ordered in terms of population. Three of the herds with positive reactors to *Brucella* (75%) came from herds whose owners also keep other livestock species, and even though it is not statistically significant, sheep and goat herds belonging to owners who also keep other livestock species have 5 times more odds of getting brucellosis compared to other herds whose owners do not keep other livestock species as well (OR = 5.045; 95% CI: 0.494–51.540, *p* = 0.172).

Nearly all of the herds 62/63 (98.4%) did not have a calving/lambing/kidding pen where animals delivered offspring, and all the herds with positive reactors to *Brucella* were from those herds that lacked the calving pen, but this was not statistically significant (*p* > 0.05). Since the area is predominantly pastoralist with grazing lands shared communally, there is a high chance of contact between different herds. In the present study, 52/63 (82.5%) of the herds came into contact with other herds of sheep and goats in the past year, but the current study did not find a statistically significant association between *Brucella* herds seropositivity and contact with other herds (OR = 0.612; 95% CI: 0.058–6.505, *p* = 0.684).

On the other hand, 14/63 (22%) of the herds came into contact with wild animals in the past year. The most commonly encountered wild animals were antelopes such as dik-dik, warthogs, foxes, and hyenas. Although it was not statistically significant, herds that came into contact with wild animals had a higher chance of having positive reactions to *Brucella* (OR = 3.917; 95% CI: 0.499–30.728, *p* = 0.194).

Abortion in sheep and goat herds was reported in 32/63 (50.8%) of the herds in the past year. Of the 4 herds that tested seropositive for *Brucella*, 3 (75%) had experienced abortion in the past year compared to 29 (49%) in seronegative herds. Herds with abortion reports in the past year had an approximately 3 times higher chance of containing seropositive reactors than herds that did not experience abortion in the past year, but this difference was not significant (OR = 3.103; 95% CI: 0.305–31.580, *p* = 0.339).

New animals were introduced into 14/63 (22.2%) of the herds in the past year mainly through purchases and gifts from relatives. Comparing herds that introduced a new animal into the herds in the past year to those herds that did not, the study found that herds that introduced new animals into the herds had 13 times increased chances of having seropositive reactors for *Brucella* infection (OR = 13.091; 95% CI: 1.241–138.11, *p* = 0.032).

Sharing of breeding males is less common, and only 13/63 (20.6%) of the herds have lent their breeding male sheep or goat animals over the last year. Herds that lend male animals for breeding purposes have a more than 14-fold increased chance of having seropositive reactors compared to herds that do not lend their male animals to other herds for breeding. This was statistically significant (OR = 14.7; 95% CI: 1.384–156.179, *p* = 0.026).

Although 26/63 (41.3%) of the herds were vaccinated in the past year, no vaccination was given against brucellosis, and only 19 (30%) respondents had received/obtained veterinary advice last year, with the majority, 44 (70%), not getting veterinary advice of any kind in the past year. No statistically significant difference (*p* > 0.05) in herds’ seropositivity was observed between the herds in relation to vaccination and receiving veterinary advice (Table 4).

**Table 4:** Univariate analysis comparison of factors associated with *Brucella* seropositivity among positive and negative herds.

Variable	Category	Herds positive, n (%)	Herds negative n (%)	OR (95% CI)	*p*
Herds’ size	>60	3 (75)	18 (30.5)	6.833 (0.665–70.235)	0.106
	≤60	1 (25)	41 (69.5)		
Ownership of other livestock species	Yes	3 (75)	22 (37.3)	5.045 (0.494–51.540)	0.172
	No	1 (25)	37 (62.7)		
Contact other sheep and goat herds in the past year	Yes	3 (75)	49 (83)	0.612 (0.058–6.505)	0.684
	No	1 (25)	10 (17)		
Contact with wild animals	Yes	2 (50)	12 (20.3)		
	No	2 (50)	47 (79.7)	3.917 (0.499–30.728)	0.194
Abortion in sheep and goat herds in past year	Yes	3 (75)	29 (49)		
	No	1 (25)	30 (51)	3.103 (0.305–31.580)	0.339
Introduction of new sheep and goats	Yes	3 (75)	11 (18.6)	13.09 (1.241–138.11)	0.032*
	No	1 (25)	48 (81.4)		
Lend breeding male in past year	Yes	3 (75)	10 (17)	14.7 (1.384–156.179)	0.026*
	No	1 (25)	49 (83)		
Seek veterinary service in past year	Yes	2 (50)	17 (29)	0.405 (0.53–3.111)	0.385
	No	2 (50)	42 (71)		

*Indicates variables with a statistically significant values. OR, odds ratio; CI, confidence interval.

#### 3.3.3. Multivariable analysis to determine independent factors associated with *Brucella* herds seropositivity

Based on the entry criteria stated in the methodology (*p* ≤ 0.25), herds’ size, ownership of other livestock species, contact with wild animals in the past year, the introduction of new animals in the past year, and lending of breeding males in the past year were selected for the multivariable analysis using a binary regression model. Additionally, age category, previous history of abortion, number of parity and retained fetal membrane history were selected for multivariable regression model from individual seropositive animal level factors. Nevertheless, none of the variables showed a significant association (*p* > 0.05) in the multivariable analysis (Table 5).

**Table 5:** Multivariable logistic regression analysis of the variables associated with herds and individual-level seropositivity for *Brucella* in sheep and goats.

Variable characteristics	OR	[95% CI]	S.E.	*p*
Herds size (>60 or ≤ 60)	5.307	0.237–118.695	1.586	0.292
Ownership of other livestock species (yes or no)	2.906	0.186–45.447	1.403	0.447
Contact with wild animals in past year (yes or no)	4.225	0.218–82.060	1.514	0.341
Introduction of new animals in past year (yes or no)	3.354	0.170–66.232	1.522	0.427
Lend breeding male in past year (yes or no)	13.398	0.714–251.590	1.496	0.083
Age category (≤2 years/3–4 years/>4 years)	18.271	0.256–1303.684	39.783	0.182
Number of parity (0–2 times, 3–4 times, 5–6 times)	24.936	0.482–1290.939	50.215	0.110
Previous history of abortion (yes or no)	4.127	2.231–19.021	1.091	0.651
Retained fetal membrane (yes or no)	2.349	0.346–12.902	1.439	0.498

OR, odds ratio; SE, standard error; CI, confidence interval.

## 4. Discussion

In this study, the animal-based seroprevalence of small ruminant brucellosis in three districts of the Korahey zone of Somali regional state was 1.23% and this was in line with the results reported by Mohammed et al. (19) in the Jigjiga zone of the Somali region, with a prevalence of 1.37%; Tsegay et al. (28) in Debrezeit and Modjo, with a seroprevalence of 1.76%; and Dabassa et al. (29), who found a similar result (1.56%) in a study of small ruminant brucellosis in Yabello. The present finding is lower than results reported by Aloto et al., (30) who reported 4.1% in two zones of southern Ethiopia, Dosa et al. (31) who reported 3.33% in two selected districts of Wolaita Zone southern region, Teshome et al. (32) who reported 17.36% in goats in Borana pastoral area, Deddefo et al. (33) who reported 4.6% in Arsi, Teshale et al. (34) who reported 9.7% in Afar, and Negash et al. (35) who reported 9.11% in the Dire Dawa area. Similarly, Wedajo et al. (18) reported a higher seroprevalence (13.7%) in the Tallalak district of the Afar region.

However, the current result is relatively higher than the previous study by Ferede et al. (36) who reported 0.4% in and around Bahirdar and Tewodros and Dawit (37) who reported 0.7% in and around Kombolcha, Amhara region. This variation might be caused by variations in sample size, agroecological location, and animal management practices.

In the present study, RBPT was used to detect the seroprevalence of *Brucella* species in small ruminants which was used in this study to screen individual animals, is a low-cost, quick, and highly sensitive test (27). However, due to cross-reactivity with antibodies from closely related gram-negative bacteria such as *Yersinia enterocolitica, Escherichia coli, Salmonella* spp., and *Sternotrophomonas maltophilia* as well as antibodies produced by the *B. abortus* S19 vaccine, its specificity is low (38). In the current study, only samples that gave signals for both RBPT and CFT were considered positive since no single test is appropriate in all epidemiological situations due to problems of sensitivity and or specificity of the tests as recommended by OIE (27) and other reports (39).

The current study revealed that the seroprevalence of brucellosis was 1.57 times higher than sheep’s seroprevalence, although this difference was not statistically significant. This finding is comparable to that of Mohammed et al. (19), who also reported higher seroprevalence in goats in the Somali region. Similar findings were also reported by Wedajo et al. (18), Teshale et al. (34), and Ashenafi et al. (40) in the Afar region; Aloto et al. (30) in two zones of southern, Ethiopia; Mengistu (41) in Konso, southern Ethiopia; Tewodros and Dawit (37) in Kombolcha of the Amhara region; and Negash et al. (35) in Dire Dawa.

However, a study by Bekele and Kasali (42) in the central highlands of Ethiopia and Samaha et al. (43) in Egypt showed a higher prevalence in sheep than in goats, mainly due to differences in husbandry systems and the susceptibility of the sheep and goat breeds in the particular area. The difference may also be due to variations in the species breakdown of the samples examined by the various researchers. Because goats are more susceptible to *Brucella* infection than sheep and excrete the bacterium for a longer period of time, goats exhibit higher seroprevalence than sheep (21).

The seroprevalence of brucellosis of female sheep and goats were higher than males one, although this difference was not statistically significant (*p* > 0.05). The result of this study is in agreement with other findings by Tewodros and Dawit (37) and Yesuf et al. (44), who also reported higher seroprevalence in females than in males, although Yesuf et al. (44) found a statistically significant (*p* < 0.05) difference in *Brucella* seroprevalence between the two sexes. The lower number of males (*n* = 48) sampled and tested compared to a higher number of females (*n* = 276) in the sample may have contributed to the higher female animal seropositivity. It is also possible that male animals are less likely to contract *Brucella* infection because they do not contain erythritol (45). The fact that there was no statistically significant difference between the two sexes may also have contributed to the very low number of positive results observed in the current study.

The study revealed a significantly (*p* < 0.05) higher seroprevalence of small ruminant brucellosis in the old age group than in the medium and young age groups. This is consistent with the findings of Tsegaye et al. (28) and Adugna et al. (46), who also reported a higher seroprevalence of brucellosis in small ruminants more than 2 years of age than in the younger age categories. Similarly, Megersa et al. (14), Mohammed et al. (19), and Tigist et al. (47) reported higher seroprevalence in older age groups than in younger animals, even though the difference was not statistically significant. In contrast, Wedajo et al. (18) and Negash et al. (35) reported a higher seroprevalence in younger animals than in adult sheep and goats.

The study also found a statistically significant association between seroprevalence and parity stage, with higher seropositivity in higher parity stage females than in lower parity stage females. The various findings regarding the variation in brucellosis seroprevalence among the various age groups may be related to variations in the relative proportion of the various age groups in the samples examined by the various researchers. The risk of contracting *Brucella* infection is higher in sexually mature and pregnant animals than in sexually immature animals of either sex. This might be due to the concentration of erythritol and sex hormones, which promote the growth and reproduction of *Brucella* species organisms, rising with age and sexual maturity (48).

In the present study, the relationship between *Brucella* seropositivity and the presence of reproductive issues, such as a history of abortion or retained fetal membranes, was investigated. Male animals of both species were excluded from this discussion because no male animals tested positive for *Brucella* infection by chance. The seroprevalence of brucellosis in small ruminants with a history of abortion or retained fetal membranes was found to be higher (*p* < 0.05) than in those without these problems. Similar findings were reported by Wedajo et al. (18) and Wubishet et al. (49) in the Afar and Guji zones of the Oromia region, respectively. It is known that abortion in livestock represents the major complaint attributed to *Brucella* infections (50–52).

The seroprevalence of brucellosis was higher in large (>60 heads) herds’ sizes than in small (≤60 heads), but the difference was not significant. This is in line with the findings reported by Wedajo et al. (18) in the Afar region. Walker (53) shows that herd sizes and animal densities are directly related to disease prevalence and complicate infection control in a population. Similarly, sheep and goat herds kept alongside other livestock species had higher brucellosis seroprevalence than herds kept solely, where the owner did not own any other livestock species besides sheep and goats.

The introduction of new animals from unscreened herds into sheep and goat herds was a major risk factor observed in this study. Pastoralists usually introduce these animals into the herds as replacement stock through purchases, gifts, or donations from relatives. This finding is consistent with the findings of several authors who discovered that the introduction of animals from non-free Brucellosis herds or herds with unknown Brucellosis status was a major factor associated with Brucellosis in sheep, goat, and cattle herds (50, 54–57).

Other research suggests that introducing infected animals can increase individual-level prevalence because the longer they are in contact with the rest of the herds, the greater the risk of spread (1, 58). Animal movement between herds has also been found to be a potentially dangerous practice. One suggested key preventive measure is to avoid the introduction of infected animals by maintaining completely closed herds or by carefully screening purchased animals before introducing them into the herds, a practice that is very uncommon in pastoral communities. There is evidence that one of the main causes of most brucellosis control campaigns’ ineffectiveness is the lack of control over the movement of animals, and this suggestion is supported by available data (12, 50, 56).

Seroprevalence was higher in herds with female animals that had abortions in the previous year compared to herds without abortions, but this difference was not statistically significant. Tigist et al. (47) and Obonyo (59) revealed a statistically significant correlation between a herd’s seropositivity to *Brucella* and the presence of female animals that had recently given birth. Brucellosis causes late-term abortions, which increases the risk of the disease spreading to other animals in the herds while they graze on the contaminated pasture lands because aborting animals typically shed the bacteria into the environment. Abortion represents the major complaint attributed to *Brucella* infections in livestock (4, 50–52, 60). It is known that females infected with brucellosis shed considerable amounts of the pathogen in milk, placental membranes, and aborted fetuses. Such females have been reported to shed organisms for several months (4, 48, 60). This causes environmental contamination, which increases the risk of pathogen transmission between animals in the same herds as well as other herds during free mixing in grazing and watering areas.

Lending or sharing male animals with other herds for natural breeding purposes was significantly associated with herd seropositivity. Other authors have reported that lending male animals for breeding is a risk factor for *Brucella* seropositivity in animals (59, 61). Although the venereal route is not regarded as a key channel for *Brucella* transmission in small ruminants under natural settings, procedures that entail the movement of animals between herds s are deemed problematic because of the possibility of mechanical transmission (5, 12, 48).

Although not significant, seropositivity was higher in herds that came into contact with other herds in the past year. This could be attributed to the pastoral lifestyle, which is characterized by the frequent mobility of herds. Considering the contagious nature of *Brucella* spp. sharing shared grazing areas and drinking holes makes it easier for possibly infected cows and clean herds to spread infections such as brucellosis and others (14, 62).

The current study was limited to the seroprevalence of the small ruminant brucellosis only and did not include other such as cattle and camels are susceptible to brucellosis and both species are kept in the study area but they were not part of the current study. The study also did not attempt to assess the prevalence level of the disease in humans to correlate findings in the animals. The present study did not attempt culture of *Brucella* species and therefore was not able to identify the various species and biovars of *Brucella* species circulating in sheep and goats in the study area.

## 5. Conclusion

The present study revealed that the seroprevalence of brucellosis in sheep and goats was found to be relatively low at both the individual animal and the herd level. However, the multivariable logistic regression analysis revealed that none of the proposed risk factors were not significantly associated with *Brucella* seropositivity at individual and herd level. In conclusion, awareness campaign among pastoral community of the seriousness of the causes, modes of transmission, symptoms, risk factors, and methods of prevention of the disease should be undertaken as soon as possible. For effective control of brucellosis that may be present in the area, an integrated approach should be promoted that takes into account the relationship between humans, animals, and the environment in the context of “One Health approach.”

## Data availability statement

The raw data supporting the conclusions of this article will be made available by the authors, without undue reservation.

## Ethics statement

The animal studies were approved by the Jigjiga University, College of Veterinary Medicine-Research Review Committee (Protocol No. JJU/CVM/clis/022/14). The studies were conducted in accordance with the local legislation and institutional requirements. Written informed consent was obtained from the owners for the participation of their animals in this study.

## Author contributions

AMH, FA, AHH, AM, and HG contributed to data collection, study design, and interpretation, manuscript draft, and writing. AMH, FA, and AHH contributed to the conception of the research idea, study design, data analysis, writing, revising and editing, the design of the study, and data interpretation. AM and HG contributed to the reference search and manuscript writing and editing. All authors approved the submission of the final manuscript.

## Funding

This study was funded by the Jigjiga University Research and Community Service Vice President Office.

## Conflict of interest

The authors declare that the research was conducted in the absence of any commercial or financial relationships that could be construed as a potential conflict of interest.

## Publisher’s note

All claims expressed in this article are solely those of the authors and do not necessarily represent those of their affiliated organizations, or those of the publisher, the editors and the reviewers. Any product that may be evaluated in this article, or claim that may be made by its manufacturer, is not guaranteed or endorsed by the publisher.

## Abbreviations

BOFED: Bureau of Finance and Economic Development
CAT: Card agglutination test
CFT: Complement fixation test
CFSPH: Center for Food Security and Public Health
ELISA: Enzyme-linked immunosorbent assay
ICSP: International Committee on Systematics of Prokaryotes
LPDB: Livestock and Pastoralist Development Bureau
NVI: National Veterinary Institute
OIE: Office International des Epizooties
RBPT: Rose Bengal Plate Test
SRBC: Sensitized sheep red blood cells
SRS: Somali regional state
WHO: World Health Organization
